# Iron changes within infarct tissue in ischemic stroke patients after successful reperfusion quantified using QSM

**DOI:** 10.1007/s00234-024-03444-6

**Published:** 2024-08-22

**Authors:** Victoria Mercy Kataike, Patricia M. Desmond, Christopher Steward, Peter J. Mitchell, Christian Davey, Nawaf Yassi, Andrew Bivard, Mark W. Parsons, Bruce C.V. Campbell, Felix Ng, Vijay Venkatraman

**Affiliations:** 1 Department of Radiology, The University of Melbourne, Parkville, VIC 3050 Australia; 2 Department of Medical Imaging, The Royal Melbourne Hospital, Parkville, VIC Australia; 3 Department of Medicine, The Royal Melbourne Hospital, University of Melbourne, Parkville, Victoria Australia; 4 Department of Neurology, Royal Melbourne Hospital, Parkville, VIC Australia; 5 Statistical Consulting Centre, School of Mathematics and Statistics, The University of Melbourne, Parkville, VIC Australia; 6 Population Health and Immunity Division, The Walter and Eliza Hall Institute of Medical Research, Parkville, Victoria Australia; 7 Hunter Medical Research Institute, Newcastle, New South Wales Australia; 8 Department of Neurology, University of New South Wales Southwestern Sydney Clinical School, Ingham Institute for Applied Medical Research, Liverpool Hospital, Sydney, New South Wales Australia

**Keywords:** Ischemic stroke, Iron, Infarct tissue, Successful reperfusion, Quantitative susceptibility mapping

## Abstract

**Purpose:**

For nearly half of patients who undergo Endovascular Thrombectomy following ischemic stroke, successful recanalisation does not guarantee a good outcome. Understanding the underlying tissue changes in the infarct tissue with the help of biomarkers specific to ischemic stroke could offer valuable insights for better treatment and patient management decisions. Using quantitative susceptibility mapping (QSM) MRI to measure cerebral iron concentration, this study aims to track the progression of iron within the infarct lesion after successful reperfusion.

**Methods:**

In a prospective study of 87 ischemic stroke patients, successfully reperfused patients underwent MRI scans at 24-to-72 h and 3 months after reperfusion. QSM maps were generated from gradient-echo MRI images. QSM values, measured in parts per billion (ppb), were extracted from ROIs defining the infarct and mirror homolog in the contralateral hemisphere and were compared cross-sectionally and longitudinally.

**Results:**

QSM values in the infarct ROIs matched those of the contralateral ROIs at 24-to-72 h, expressed as median (interquartile range) ppb [0.71(-7.67-10.09) vs. 2.20(-10.50-14.05) ppb, *p* = 0.55], but were higher at 3 months [10.68(-2.30-21.10) vs. -1.27(-12.98-9.82) ppb, *p* < 0.001]. The infarct QSM values at 3 months were significantly higher than those at 24-to-72 h [10.41(-2.50-18.27) ppb vs. 1.68(-10.36-12.25) ppb, *p* < 0.001]. Infarct QSM at 24-to-72 h and patient outcome measured at three months did not demonstrate a significant association.

**Conclusion:**

Following successful endovascular reperfusion, iron concentration in infarct tissue, as measured by QSM increases over time compared to that in healthy tissue. However, its significance warrants further investigation.

**Supplementary Information:**

The online version contains supplementary material available at 10.1007/s00234-024-03444-6.

## Introduction

Stroke ranks second globally in causes of death and third in causes of disability [1]. Endovascular thrombectomy (EVT) is the standard treatment for patients with acute large vessel occlusions, yet up to half of the treated patients still experience poor long-term results, termed futile recanalization or clinically ineffective reperfusion, so understanding why this occurs is crucial [2, 3].

Brain tissue affected by ischemic stroke has traditionally been viewed as completely salvaged by timely reperfusion or completely damaged within the infarct. However, recent histological and imaging studies have challenged this dichotomy and presented a spectrum of tissue fate: (1) remote benign oligemia without tissue dysfunction, (2) salvaged penumbra tissue with neuronal dysfunction without sustained structural injury, admixed in varying proportions with (3) regions of irreversible tissue destruction [4]. The size and extent of these regions depend on the severity and duration of the initial ischemic insult.

Iron is an abundant trace material in the brain, essential for cellular key functions such as the production of ATP, DNA synthesis, myelin synthesis, mitochondrial respiration, and cell metabolism [5, 6]. However an imbalance in iron, either in deficiency or excess has detrimental effects on brain health [7].

Excess iron accumulation is associated with several neurodegenerative diseases like Parkinson’s and Alzheimer’s [8]. In the context of ischemic stroke, iron homeostasis breaks down on the onset of cerebral ischemia leading to the abnormal accumulation of iron in tissue. This iron accumulation triggers reactions that worsen tissue damage, contributing to the severity of ischemic stroke [9, 10]. Iron has also been implicated in ischemia/reperfusion injury, where the sudden restoration of blood flow to affected tissue paradoxically exacerbates tissue damage [11]. Given the significant involvement of iron in ischemic stroke pathology, it could serve as a potential biomarker for understanding the underlying causes and effects of the differential outcome seen in stroke patients after successful reperfusion.

Iron distribution in the brain can be visualised non-invasively using MRI techniques sensitive to magnetic susceptibility. These techniques include T2-weighted imaging, T2* (or R2*) mapping, susceptibility-weighted imaging (SWI), and recently, quantitative susceptibility mapping (QSM) [5, 6]. QSM is a technique that quantifies the spatial distribution of magnetic susceptibility within tissue. Langkammer et al. [12] found that iron is a dominant contributor to the magnetic susceptibility signal, thus QSM provides an indirect measure of brain iron concentration.

QSM has been employed in understanding neurodegenerative disorders like Parkinson’s and Alzheimer’s where a high concentration of iron has been linked to the occurrence of these disorders. In the context of ischemic stroke, QSM has been used to study oxygen metabolism, as well as vascular and tissue changes in animal stroke models and patient cohorts. Cross-sectional and longitudinal differences in magnetic susceptibility were found between infarct and healthy regions of the brain [5, 13–17]. Despite the above findings, limited investigations have delved into the longitudinal QSM changes specifically in infarct tissue, particularly in patients who have undergone successful reperfusion.

Thus, the purpose of this study is to examine the longitudinal changes in iron concentration as quantified by QSM in infarct tissue and healthy tissue in a patient cohort of successfully reperfused patients. The longitudinal nature of the study from the acute stage of ischemic stroke to the chronic stage of ischemic stroke spans a much longer period than previous research allowing for a comprehensive assessment of QSM changes. In studying the QSM changes, we can obtain insights on the underlying infarct tissue changes shown by changes in the concentration of iron in patients who have been successfully treated and are expected to have a better recovery [12].

## Materials and methods

### Patient information

The patients in this study participated in the prospective observational PRAISE (Post Reperfusion pathophysiology in Acute Ischemic StrokE) study, 2018–2022, Trial Registration Number - ACTRN12624000629538, which performed serial multiparametric MRI on 87 patients treated with reperfusion therapy for anterior circulation large vessel occlusion at two Comprehensive Stroke Centres (80 patients from Stroke Centre I and 7 patients from Stroke Centre II). The local Human Research Ethics Committee of the participating institutes approved the study. Informed consent was given before participating in the study.

The patients were scanned using MRI at four time points after reperfusion: immediately after EVT, 24-to-72 h, 3 months, and 12 months, with QSM sequences performed at the latter three time points. NIHSS was recorded at admission and immediately after EVT, while functional outcome was assessed using mRS at 3 months and 12 months.

### Acquisition of magnetic resonance imaging data

Patients were scanned on a 3T Siemens Prismafit scanner. The scanning protocol was; DWI-Resolve (b-value = 1000 s/mm^2^, TR = 5100 ms, TE = 55 ms, Resolution = 0.98 × 0.98 × 4 mm^3^, Acquisition time = 2 mins 19 s), T2 FLAIR (TR = 6700 ms, TE = 463 ms, TI = 2200 ms, Resolution = 1 × 1 × 1 mm^3^, FA = 120 °, Acquisition time = 5 min 4 s), and 3D multi-echo gradient echo (TR = 50 ms, 9 echoes TE = 5.84 ms − 44.16 ms with a spacing of 4.79 ms, FA = 15, GRAPPA with acceleration factor 3, Pixel bandwidth = 310 Hz, Flow compensated, Acquisition time = 8 min 4 s). DWI and GRE images were obtained at the 24-to-72 h after reperfusion, while T2 FLAIR and GRE images were obtained at 3 months and 12 months.

QSM maps were generated from the gradient echo images using the Morphology Enabled Dipole Inversion (MEDI) pipeline available at https://pre.weill.cornell.edu/mri/pages/qsm.html. Standard parameters of the MEDI pipeline were used. Briefly, phase unwrapping was done by a region-growing spatial phase unwrapping technique, followed by brain mask extraction using Brain Extraction Tool (BET) from the FMRIB Software Library (FSL) [18]. Removal of the unwanted background field was performed using Projection onto Dipole Fields (PDF) [19]. Dipole inversion was then performed using Morphology Enabled Dipole Inversion with zero reference, where the susceptibility measurements were normalized to Cerebrospinal Fluid (CSF) measurements (MEDI + 0) [20].

### Image processing

All images were skull-stripped using the FreeSurfer MRI synthstrip function [21]. DWI and FLAIR images were co-registered to QSM space using FSL-FLIRT [22], with manual verification.

**Fig. 1:**
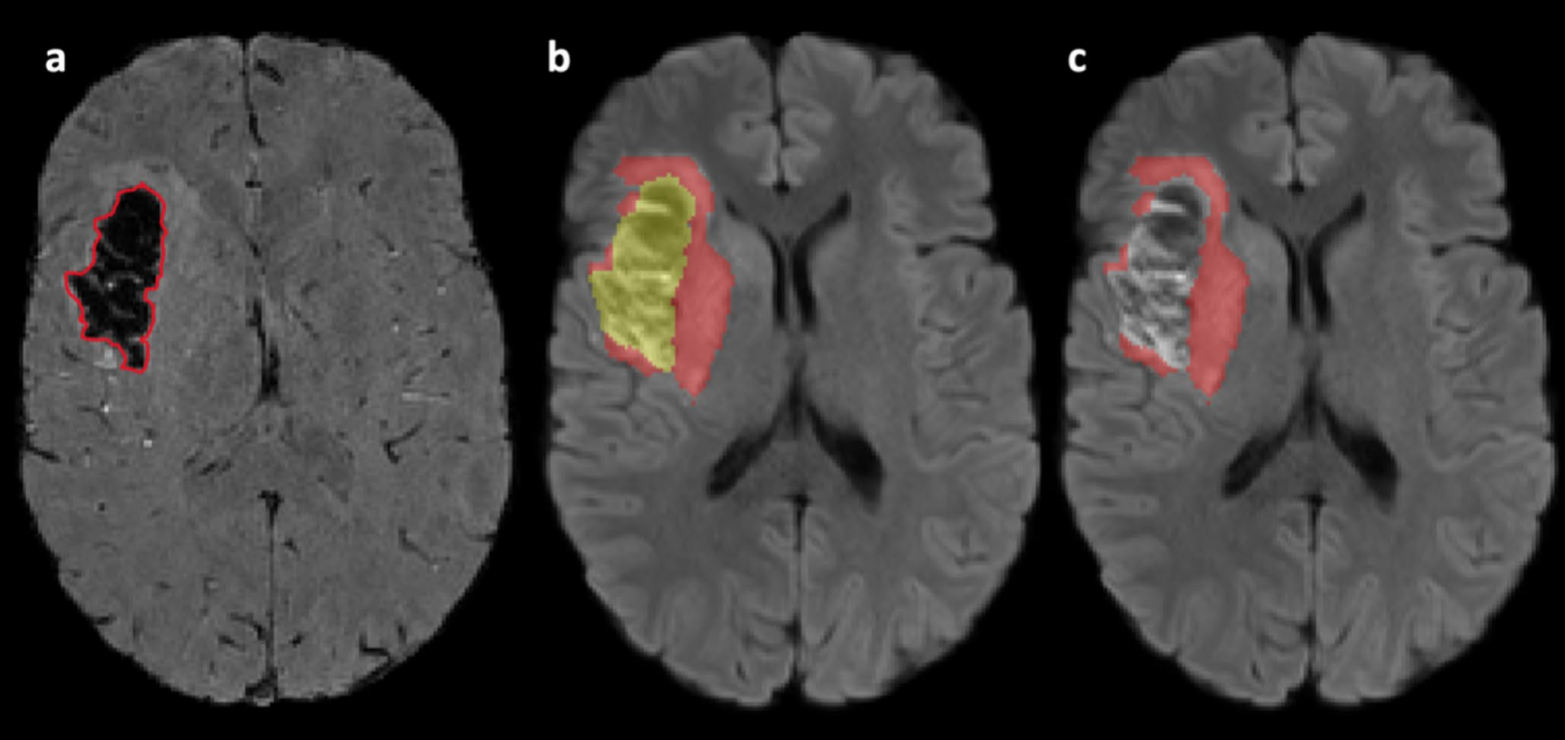
Haemorrhage elimination. The hypointense region on the magnitude image of the sixth echo of the GRE images was delineated to create a mask (**a**). In (**b**) the mask from (**a**) was applied to the infarct ROI (red), to produce a haemorrhage-free infarct ROI, shown in (**c**)

**Fig. 2:**
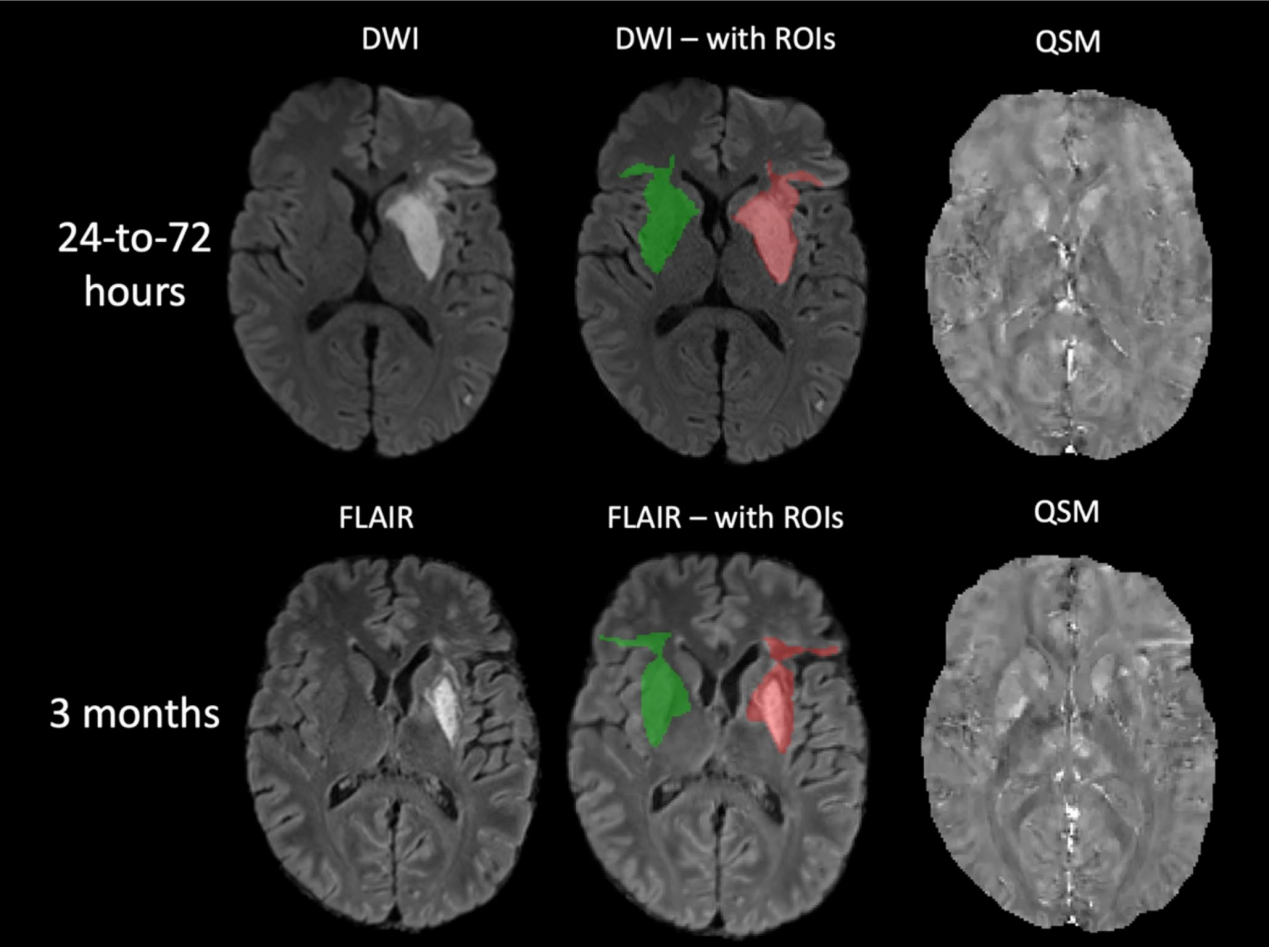
Representative DWI, FLAIR, and QSM images of a 55-year-old male with an M1 occlusion-type ischemic stroke. ROIs indicate the infarct (red) and the contralateral regions (green). DWI = diffusion-weighted imaging, FLAIR = fluid-attenuated inversion recovery, QSM = quantitative susceptibility mapping

An expert neuroradiologist (30 years’ experience) manually delineated infarct regions on DWI images (24-to-72 h after reperfusion) and T2 FLAIR (3 months). Automated delineation of corresponding mirror homologs in the contralateral hemisphere was done using a pipeline containing flirt, convert_xfm and fslswapdim commands from FSL. At each time point, ROIs were manually edited using ITK-SNAP to remove CSF regions [23]. Regions of haemorrhage within the infarct were identified at the 24-to-27 hours’ time point using the magnitude image of the sixth echo to balance between T2* weighting and the blooming effect. If hypointense regions within the infarct were identified, indicating the presence of haemorrhage sites, they were manually outlined and then removed from the infarct tissue ROIs at all time points using various commands from FSL (Fig. 1) [24]. After removing CSF and haemorrhages, the final ROIs were applied as masks to the QSM images, and the mean QSM values were obtained in parts per billion (ppb) (Fig. 2).

The datasets used and/or analysed during this study are available upon reasonable request from the corresponding author.

### Statistical analysis

Data normality was tested using the Shapiro-Wilk test. Normally distributed continuous variables are presented as mean ± standard deviation; non-normally distributed variables are presented as median (interquartile range), and categorical variables as absolute frequencies. IBM SPSS version 28.0.1.1 and R version 4.2.3 were used for statistical analyses.

The Wilcoxon signed-rank test was employed for the cross-sectional comparison of QSM values in the infarct and contralateral ROIs at each time point using the whole dataset in SPSS. Furthermore, QSM values in the infarct and contralateral ROIs were compared longitudinally using the Wilcoxon signed-rank test in SPSS on the dataset with both time points.

The association between infarct QSM at 24-to-72 h and mRS at 3 months was investigated using ordinal logistic regression analysis with age, sex, and infarct volume at 24-to-72 h as covariates. A similar analysis was conducted with infarct QSM at 3 months.

We performed a linear mixed model analysis to evaluate the effect of various patient characteristics on infarct QSM values at both time points. Presence of a haemorrhaging in the infarct, age, sex, infarct volume, presenting NIHSS and time were set as the fixed effects. Patient ID was set as the random effect to account for within–patient variability. Statistical significance was set at *p* < 0.05 for all tests.

## Results

### Patient characteristics

A Mann-Whitney test performed to assess for differences in patient characteristics between the participants scanned at Stroke Centre A and Stroke Centre B showed no differences in age (*p* = 0.146), NIHSS (*p* = 0.22) and mRS scores at 3 months (*p* = 0.23) between the two patient groups.

Patients with successful EVT procedures, defined as eTICI score of 2b, 2c, and 3 [25] were included in the study. Out of the 87 initially recruited patients, four were excluded due to unsuccessful EVT (eTICI = 0-2a), and seven were excluded due to rapid clinical improvement after pre-thrombectomy thrombolysis and did not proceed to EVT. Another eight were excluded due to incomplete post-thrombectomy imaging or suboptimal image quality, resulting in 68 patients with interpretable MRI results at least at one time point. The final number for QSM analysis was 64 patients at 24-to-72 h and 43 patients at 3 months, with 39 having complete data at both time points. Table 1 summarises the characteristics of the entire cohort, at the different time points, and of the 39 patients who had complete imaging data at both time points. To address potential selection bias due to attrition of patients over time, Mann-Whitney U-tests and chi-square tests were performed to compare differences in baseline characteristics between the entire cohort of 87 patients and the 43 patients at 3 months (Table 1).

**Table 1:** Patient characteristics

Patient Characteristics	Whole cohort (87 patients)	24-to-72 h (64 patients)	3 months (43 patients)	Patients with imaging at both time points (39 patients)	Selection bias Whole cohort vs. 3 months (*p*-value)
Age in years, median (IQR)	72(58–79)	73(59–80)	68(55–74)	68(55–74)	0.144
Sex	F = 37 M = 50	F = 27 M = 37	F = 19 M = 24	F = 18 M = 21	0.858
Presenting NIHSS, median (IQR)	16(11–20)	16(11–20)	15(11–20)	15(10–19)	0.794
NIHSS post EVT	8(3–12)	8(3–11)	6(2–10)	7(2–10)	0.128
mRS at 3 months, median (IQR)	2(1–4)	2(1–3)	1(1–2)	1(1–2)	0.127
Stroke localisation	Left = 40 Right = 47	Left = 28 Right = 36	Left = 23 Right = 20	Left = 21 Right = 18	0.420
Thrombolytic administered	Yes = 41 No = 46	Yes = 29 No = 35	Yes = 20 No = 23	Yes = 17 No = 22	0.947
Occlusion type	M1 = 59 M2 = 18 ICA = 9 NR = 1	M1 = 45 M2 = 12 ICA = 7	M1 = 30 M2 = 6 ICA = 7	M1 = 26 M2 = 6 ICA = 7	0.564
Reperfusion status, TICI score	0 = 2 2a = 2 2b = 40 2c = 9 3 = 27 NR = 7	2b = 30 2c = 8 3 = 26	2b = 20 2c = 4 3 = 19	2b = 17 2c = 4 3 = 18	0.230
Time from onset to CT in hours, median (IQR)	2.52(1.62–5.41)	2.67(1.72–5.55)	3.18(1.62–6.09)	2.98(1.70–5.43)	0.816
Time from onset to reperfusion in hours, median (IQR)	5.18(3.68–7.81)	5.73(3.90–7.75)	5.74(3.72–7.84)	5.73(3.90–7.43)	0.794
Time from reperfusion to MRI at 24-to-72 h in hours, median (IQR)	47.18(27.33–70.40)	46.89(27.62–70.13)	46.43(27.21–61.77)	46.28(27.19–58.51)	0.703

median (IQR) = median (interquartile range), NIHSS = National Institutes of Health Stroke Scale, mRS = modified Rankin Scale, TICI = Thrombolysis in Cerebral Infarction score, NR = not recorded

We investigated for differences in QSM values in patients who had a haemorrhage and patients who did not at each time point. We used the Mann- Whitney U test to compare the QSM values of these two groups at each time point. The results showed no significant differences between the two groups regardless of time point; thus, the QSM values of both groups were merged into one group of QSM values at each time point (*p* = 0.22 at 24-to-72 h, and *p* = 0.80 at 3 months).

### Cross-sectional comparison of QSM

The QSM values at 24-to-72 h were similar between the infarct ROIs and contralateral ROIs [infarct ROI 0.71(-7.67-10.09) vs. contralateral ROI 2.20(-10.50-14.05) ppb, *p* = 0.55]. However, at 3 months, the QSM values in the infarct ROIs were significantly higher than those in the contralateral ROIs [infarct ROI 10.679(-2.30-21.10) vs. contralateral ROI − 1.27(-12.98-9.82) ppb, *p* < 0.001].

### Longitudinal comparison of QSM

**Fig. 3:**
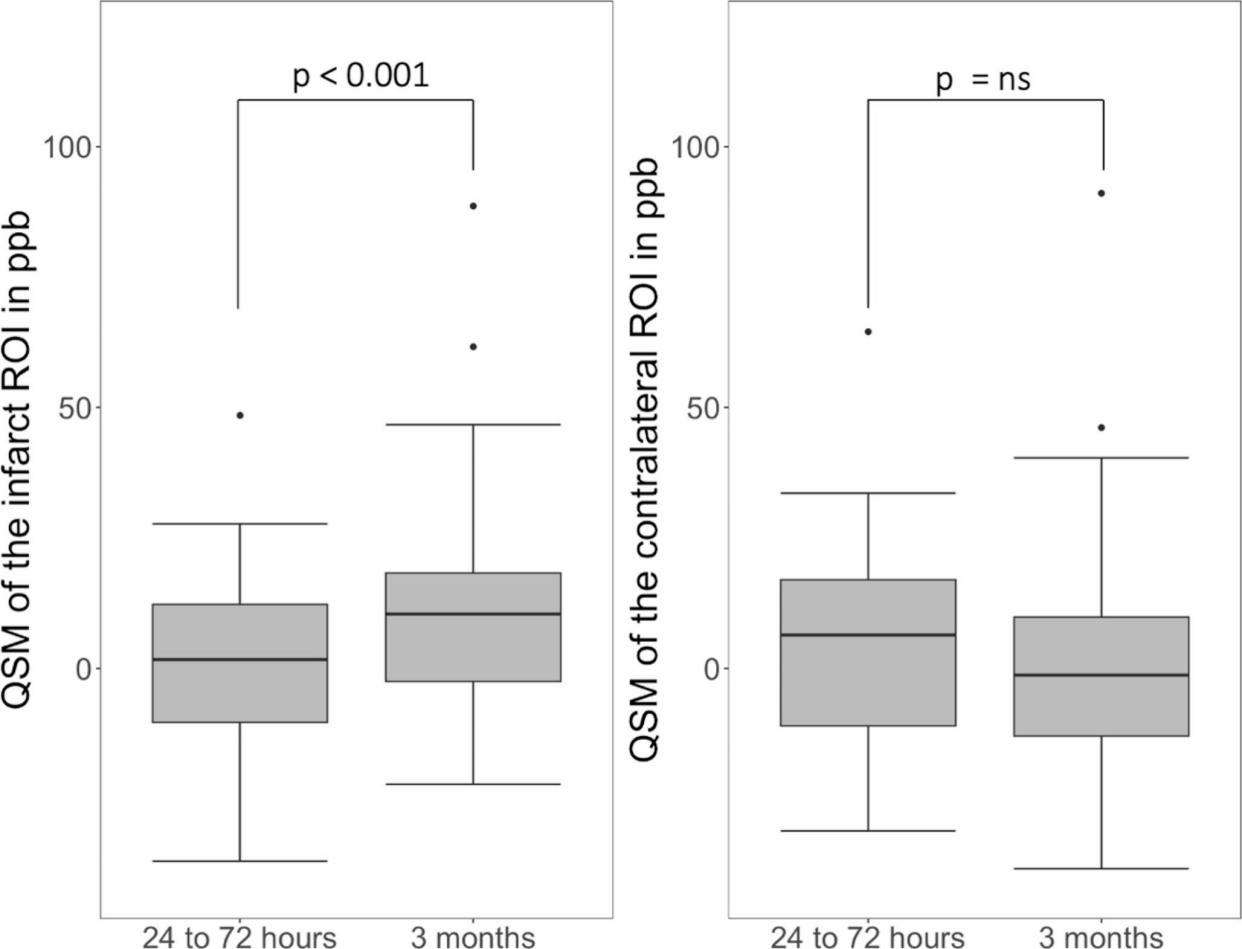
Longitudinal comparison of QSM values in infarct and contralateral ROIs. ns = not significant

In 39 patients with complete imaging data at both time points, infarct QSM values significantly increased at 3 months compared to 24-to-72 h [1.68(-10.36-12.25) ppb vs. 10.41(-2.50-18.27) ppb, *p* < 0.001] (Fig. 3). Contralateral ROIs showed no significant changes over time [6.40(-11.03-16.97) ppb vs. -1.27(-12.98-9.81) ppb, *p* = 0.270].

To address possible lesional atrophy explaining the increase in intralesional QSM over time, the correlation between infarct volume and QSM value changes was examined, revealing no significant associations between QSM change and volume change from 24-to-72 h to 3 months (Spearman’s correlation = 0.08, *p* = 0.65).

Included in the supplementary materials is a further statistical evaluation utilizing patient imaging data obtained at 12 months after reperfusion. The statistical evaluation includes the cross sectional and longitudinal analysis of the QSM values in the infarct and contralateral ROIs. An ordinal logistic regression analysis was also performed to assess for potential correlations between mRS at 12 months and infarct QSM values at 24-to-72 h, as well as 12 months.

### Regression analyses

In the ordinal logistic regression adjusted for age, sex, and infarct volume at 24-to-72 h, the infarct QSM values at 24-to-72 h showed no association with mRS at 3 months (OR = 0.58, 95%CI -1.25–0.11, *p* = 0.11).

Similarly, there was no significant association between infarct QSM values at 3 months and mRS at 3 months in the multivariate model (OR = 0.99, 95%CI -0.04–0.02, *p* = 0.59).

The results from the linear mixed model analysis showed a significant effect of time (*p* = 0.008), but not the other fixed effects – sex (*p* = 0.88), age (*p* = 0.39), infarct volume (*p* = 0.35) and presenting NIHSS (*p* = 0.76) on infarct QSM values at both time points. Analysing the different combinations of interaction effects from the fixed effects showed no statistically significant effect on the infarct QSM values.

## Discussion

This study delves into the novel investigation of the temporal evolution of the QSM metric in infarcted tissue compared to its analogous non-ischemic region in patients who had successful angiographic reperfusion, a patient cohort that has not been widely studied. Moreover, the time frame of this study spans longer than previous clinical and animal studies have investigated to provide a comprehensive understanding of QSM changes beyond the ischemic stroke event and standard patient treatment. The datasets used in this study are available upon reasonable request from the corresponding author. We showed that the amount of iron detected by QSM in infarct tissue generally increased in the radiologically defined infarct lesion over time after reperfusion. We also showed that the iron concentration in infarct tissue was higher than in contralateral tissue at later time points after reperfusion. Notably, there was no observed association between infarct QSM values at 24-to-72 h and 3 months, and patient outcome measured using mRS obtained at 3 months after reperfusion. Despite the significant increase in infarct tissue QSM values after three months and at 12 months (as per supplementary analysis), the infarct tissue QSM values at the 24-to-72 h timepoint were less than the QSM values in contralateral tissue but did not display any significant differences. Similar results were obtained in a murine model of post-ischemic reperfusion by Vaas et al. [5] and a cross-sectional clinical study by Probst et al. [14] despite the difference in methodologies employed to obtain and quantify the QSM in the infarct and contralateral regions.

Vaas et al. [5] qualitatively investigated vascular and tissue-specific changes in mice at different time points from two to 48 h after reperfusion. They observed an increase in magnetic susceptibility in prominent veins located around the core of the lesion with time, compared to those observed in the contralateral hemisphere. Magnetic susceptibility within the infarct tissue, however, was lower in the ipsilateral hemisphere than in the contralateral hemisphere, with the differences in susceptibility most apparent at 24 and 48 h after reperfusion. Vaas et al. [5] attributed the high QSM values in the prominent vessels to increased oxygen extraction in regions that are in the penumbral region of the ischemic lesion, while the low QSM values in tissue were attributed to low or absent oxygen extraction in the core region of the ischemic lesion.

Juxtaposing with our study, though we found a similar trend in tissue changes at 24-to-72 h. This trend was opposite to the trend they found in the vascular changes, probably due to our general tissue approach that encompassed several structures, including the venous structures. These different structures had different contributions to the magnetic susceptibility value [26, 27].

In the ischemic stroke patient study by Probst et al. [14], the QSM values of cortical veins in the infarct lesion were significantly less than the values of similar veins in the ipsilateral but non-ischemic territory as well as in the contralateral territory in successfully recanalized patients 24-to-72 h after reperfusion. Like the conclusions from Vaas et al. [5], the decrease in QSM values in the infarct tissue was attributed to poor oxygen extraction because of tissue damage. Though this decrease in infarct QSM in this study was similar to the current study with tissue QSM despite not reaching statistical significance, a notable shift in QSM trends emerged at the later time points in the current study, where the QSM of the infarct tissue surpassed that of the contralateral tissue. This could suggest a delayed rise in the QSM value, and consequently the concentration of iron.

A recently published article examined the longitudinal iron and myelin changes within ischemic lesions quantified by QSM and R2^*^ in ischemic stroke patients before and after stroke rehabilitation. Their results showed no significant differences in magnetic susceptibility across the patient cohort [17]. However, grouping the patients according to stroke subtypes revealed that the changes in mean magnetic susceptibility values were higher in patients with branch atheromatous disease than in other stroke subtypes. The non-significant result from the longitudinal assessment in their analysis of the whole cohort contrasts with the results from our study. This could be attributed to the different management and treatment approaches in both studies. The patients in the current study were treated with EVT while the patients in the published article received rehabilitation. Another probable reason could be because the current study included patients with one stroke subtype, that is the large vessel occlusion, while the published article included patients with different stroke subtypes. The significant difference obtained in the patients with branch atheromatous disease but not the other stroke subtypes on subgrouping highlights the distinction in results obtained in both studies.

Vaas et al. [5] and Probst et al. [14] quantified QSM differences in vascular structures, drawing conclusions based on the presence of deoxyhemoglobin in blood as the source of magnetic susceptibility. The current study took a different approach and quantified QSM values of ROIs delineating tissue, which encompassed blood vessels, neurons, glial cells, and oligodendrocytes, to examine changes within the tissue as a whole. Notably, the tissue QSM values are a summation of three magnetic susceptibility contributions; nonblood neural tissue susceptibility, plasma, and haemoglobin which depends on venous oxygenation [26]. From these three contributions, various biological materials could contribute to the susceptibility value with iron and myelin being the major contributors [27]. We were unable to distinguish between different sources of susceptibility values. As a result, we interpreted the findings with the knowledge that iron contributes the most to the QSM values we obtained. This conclusion was previously reached by Langkammer et al. [12] in their post-mortem study, where they demonstrated that iron was the primary source of magnetic susceptibility when quantified using QSM. Recent studies such as the one done by Dimov et al. [28], Chen et al. [29] and many other researchers are proposing methods to separate the diamagnetic and paramagnetic contributions to the susceptibility of a given tissue.

Grey matter and white matter tissue respond differently to ischemia, with grey matter damage resulting from neuronal loss and white matter damage from myelin damage [30, 31]. The ROIs used in the current study included both white matter and grey matter tissue and were analysed without distinguishing the two tissue types. However, in a monkey brain study conducted by Meng et al. [16], they found that the QSM in the grey matter infarct decreased over time, while the QSM in the white matter infarct increased over time. The increase in QSM values of white matter tissue was indicative of tissue damage as it is mainly composed of myelin which is diamagnetic and thus has a negative magnetic susceptibility.

Experimentally, previous studies conducted on the temporal profile of iron after ischemic stroke revealed that the harmful effects of iron released during ischemic stroke on neuronal tissue can persist and were detected up to six weeks following the stroke event [10, 32]. This could have implications on patient outcome. However, our study could not find a significant association between mRS at 3 months, or mRS at 12 months (supplementary analysis) as measures of patient outcome and the infarct QSM values at 24-to-72 h and 3 months. The small sample size of 39 patients could probably have underpowered the study thus leading to the non-significant results. However, the longitudinal study by Uchida et al. [17] observed a significant increase in the NIHSS change from the first MRI to the second MRI scan in 10 patients who were identified as having increased iron from the first to the second MRI scans, indicating less patient improvement in their neurological outcome. It is probable that the difference in how NIHSS and mRS are scored could be responsible for this outcome, despite the small patient sample of 32 patients in the previous study [33].

Our linear mixed model analysis correlating infarct QSM and fixed effects of age, sex, infarct volume and presenting NIHSS yielded no significant results. We observed a statistically significant relationship between QSM values with time which has been discussed in the previous paragraphs as the significant change in infarct QSM values over time.

Conversely, other authors have suggested that increased iron may aid in stroke recovery.

A recent mice study by Guo et al. [34] saw evidence of an increase in the level of cerebral total iron in the cortex over time, and an increase and eventual decrease in the expression of the iron storage protein, ferritin. When deferoxamine nanoparticles (known to reduce iron concentration) were injected into the mice on the 3rd day of ischemic stroke, the mice had aggravated neuronal damage and delayed neurological function recovery. Furthermore, an experiment introducing an iron-promoting agent showed that excess iron was detrimental to the recovery of the mice. The authors concluded that elevated iron, rather than excess iron may aid in stroke rehabilitation. These findings suggest there could be recovery aspects of cerebral iron in patients.

In our study, we show that there is generally an increase in iron concentration in infarct tissue over time, beyond the time frames measured in any of the previous studies. However, the impact of this iron accumulation on infarct tissue is uncertain – whether it is beneficial or detrimental. There are various sources of iron within any given tissue ROI, thus the increase in iron levels could be due to a combination of hemosiderin and ferritin within tissue, and /or higher deoxyhemoglobin within the veins in the viable regions within infarct tissue due to increased oxygen extraction [27]. This necessitates further investigations into the sources of iron that increase after successful reperfusion.

While we acknowledge the limitations of accurately measuring tissue QSM, our approach of conducting longitudinal QSM studies in tissue circumvents the difficulty of measuring changes in small vascular structures over time. Furthermore, our methodology of quantifying tissue changes after successful reperfusion using non-invasive imaging of magnetic susceptibility could pave the way for new insights into the condition of infarct tissue after patient treatment in routine clinical settings. This could have significant implications in determining the best patient management strategies for each patient to increase their chances for a better outcome.

In particular, a review by Ospel et al. [35] revealed that patients who have unfavourable clinical outcomes despite successful recanalization could benefit from post-recanalization cerebroprotection. However, several challenges have prevented the implementation of cerebroprotection trials, with limited information available on the mechanisms of tissue damage post-recanalization being a major hurdle. Our current study marks the initial step toward unravelling the underlying tissue changes that could revolutionize the trajectory of post-reperfusion patient management.

Another main limitation of the study was the fluctuating patient numbers due to patient attrition fuelled by changes in health practice during the COVID-19 pandemic. There may have been an inadvertent selection bias in favour of patients who had improved recovery and fewer symptoms compared with patients who had poor outcomes and were non-ambulant. To mitigate this, the longitudinal analysis focused on patients with consistent image availability. The linear mixed model on all data and Wilcoxon signed-rank tests also yielded congruent results. The fluctuating patient numbers meant that there were fewer patients to carry out analyses comparing QSM values with patient outcome measured by mRS, which yielded negative results.

In conclusion, our study showed that in patients who had received successful reperfusion treatment, the levels of cerebral iron increase over time in the infarct tissue. However, further studies are required to assess the sources of magnetic susceptibility responsible for the obtained QSM values and thereby correctly determine whether the increase in iron is beneficial or detrimental to these patients’ recovery. Also, by correlating these findings with other metrics like oxygen metabolism and perfusion measures, we can have more insight into other aspects of underlying tissue changes in these patients.

## Electronic supplementary material

Below is the link to the electronic supplementary material.

Supplementary Material 1
