# Supplementary material for: Iron changes within infarct tissue in ischemic stroke patients after successful reperfusion quantified using QSM
Source: Neuroradiology. 2024 Aug 22;66(12):2233–42. doi: 10.1007/s00234-024-03444-6 (PMC11611990; doi:10.1007/s00234-024-03444-6)
Supplement: Supplementary file 1 — Supplementary Material 1 [file 234_2024_3444_MOESM1_ESM.pdf]

Supplementary Information

Additional statistical analysis

An additional statistical analysis was conducted for 32 patients with complete imaging data at 12 months. The cross-sectional comparison of QSM values in the infarct and contralateral ROIs revealed significantly higher QSM values in the infarct ROIs than in the contralateral ROIs [13.81(6.10-30.71) vs 0.87(-9.96-13.43) ppb, $p = 0.001$].

The longitudinal analysis included 26 patients with complete imaging data at all three time points. Infarct ROI QSM values increased significantly over time; 24-to-72 hours: 0.27(-11.00-9.72) ppb, 3 months: 10.55(-2.73-21.25) ppb [$p = 0.002$ versus 24-to-72 hours], 12 months: 10.78(4.84-23.80) ppb [$p < 0.001$ versus 24-to-72 hours]. Though infarct ROI QSM values at 12 months were higher than those at 3 months, they did not reach statistical significance ($p = 0.20$). Contralateral ROI QSM values were similar and showed no statistical significance at 24-to-72 hours and 12 months ($p = 0.83$), as well as 3 months and 12 months ($p = 0.62$).

In the ordinal logistic regression adjusted for age, sex, and volume of the infarct, there was no association between QSM of the infarct at 24-to-72 hours with mRS at 12 months (OR = 1.00, 95%CI -0.05 – 0.05, $p = 0.92$, Table S1) or between QSM of the infarct ROIs at 3 months with mRS at 12 months (OR = 1.02, 95%CI -0.02 – 0.06, $p = 0.28$).

In addition, no association was found between the QSM of the infarct ROIs at 12 months and the mRS at 12 months (OR = 0.71, 95%CI -1.22 – 0.50, $p = 0.43$).
